# Prepregnancy Serum Creatinine Concentration and Risk of Severe Maternal Morbidity and Mortality

**DOI:** 10.1016/j.xkme.2026.101455

**Published:** 2026-06-25

**Authors:** Ziv Harel, Yuguang Kang, Maria P. Velez, Natalie Dayan, Joel G. Ray

**Affiliations:** 1 Division of Nephrology, Department of Medicine, St. Michael’s Hospital, Toronto, Ontario, Canada; 2 ICES, Ontario, Canada; 3 London Health Sciences Centre Research Institute, London, Ontario, Canada; 4 Department of Obstetrics and Gynecology, McGill University, McGill University Health Centre - Reproductive Centre, Montreal, Quebec, Canada; 5 Research Institute, McGill University Health Centre, Montreal, Quebec, Canada; 6 Department of Medicine, McGill University, Montreal, Quebec, Canada; 7 Division of Medicin, Department of Obstetrics and Gynecology, St. Michael’s Hospital, Toronto, Ontario, Canada

To the Editor:

Severe maternal morbidity and mortality (SMM-M) is a composite indicator of both maternal health and quality of obstetrical care.^1^^,^^2^ Abnormal serum creatinine (SCr) concentration thresholds measured before, during, and after pregnancy are linked to adverse maternal outcomes, including preeclampsia—1 major contributor to SMM-M.^3^^,^^4^ The association between prepregnancy SCr concentration—at both abnormally high and low levels—and subsequent SMM-M is unknown.

Using linked administrative health databases, this retrospective population-based cohort study was completed in Ontario, Canada, where universal healthcare is available to all residents. The study databases contain all in-hospital births in Ontario, with 98% successful linkage of maternal and newborn records, and prior validation of sociodemographic data, primary diagnoses, laboratory data, and physician billing claims (Tables S1 and S2).

Included were women aged 16 to 50 years with a singleton live birth at 20+ weeks’ gestation between January 2009 and March 2023, and who had at least 1 outpatient measurement of SCr concentration within 365 days before the index pregnancy conception (Table S2). Excluded were those receiving dialysis or who had a kidney transplant within 365 days before the conception date, those with an SCr concentration measured after conception, and those who died before 20 weeks’ gestation.

The study exposure was the SCr concentration, measured within 365 days before conception. If a woman had more than 1 SCr concentration, then the nearest to conception was used.

The primary study outcome was SMM-M from 20 weeks’ gestation up to 42 days after the index birth. SMM was identified using a validated composite measure developed by the Canadian Perinatal Surveillance System.^5^ The SMM composite comprises 40 measures of morbidity arising in pregnancy, during labor, or postpartum. All-cause mortality was combined with SMM to form the primary composite outcome, “SMM-M.” Modified Poisson regression, including a restricted cubic spline function, generated adjusted relative risks and 95% confidence intervals (CI). Additional analysis adjusted for body mass index as a covariate.

As not all women had a preconception SCr concentration, a comparison was made between those who did and did not have a prepregnancy SCr concentration.

A standardized difference > 0.10 was considered clinically important. Among 1,869,326 hospital singleton births in Ontario during the study period, 471,242 (25.2%) had a prepregnancy SCr concentration and were included in the final cohort (Table 1). Those who had an SCr concentration measured were slightly older and had higher comorbidity measures than those who did not have an SCr concentration measured (Table 1 and Fig S1). The mean age of the included cohort was 31 years, and the mean (SD) SCr concentration was 61.8 (10.4) μmol/L. Almost 6% of women conceived using assisted reproduction, and preeclampsia or gestational hypertension occurred among 0.8% of births.

**Table 1:** Characteristics of 1,869,326 Consecutive Singleton Hospital Births In Ontario, Including Those Who Did and Did Not Have A Serum Creatinine Concentration Measured Within 365 Days Before Pregnancy Conception

Characteristic	Serum Creatinine Concentration Was Measured (N=471,242)	Serum Creatinine Concentration Was Not Measured (N=1,305,681)	Standardized Difference^a^
Of the mother			
Mean (SD) age at conception, y	31.1 (5.0)	29.7 (5.3)	0.27
< 35	352,853 (74.9)	1,068,001 (81.8)	0.17
35-39	97,311 (20.6)	202,832 (15.5)	0.13
40-44	19,596 (4.2)	33,125 (2.5)	0.09
45-50	1482 (0.3)	1723 (0.1)	0.04
Median (IQR) parity	1 (0-1)	1 (0-1)	—
Parity ≥ 1	253,656 (53.8)	750,461 (57.5)	0.07
Residential income quintile			
1 (lowest)	98,807 (21.0)	288,720 (22.1)	0.00
2	94,700 (20.1)	264,529 (20.3)	0.00
3	100,872 (21.4)	274,094 (21.0)	0.01
4	100,407 (21.3)	265,636 (20.3)	0.02
5 (highest)	76,456 (16.2)	212,702 (16.3)	0.00
Rural residence	30,567 (6.5)	146,860 (11.2)	0.17
Maternal world region of origin			
Canada/long-term resident	296,302 (62.9)	965,514 (73.9)	0.24
Caribbean/Africa	8004 (1.7)	17209 (1.3)	0.03
East Asia/Pacific	37,877 (8.0)	82,423 (6.3)	0.07
Hispanic America	11,735 (2.5)	25,178 (1.9)	0.04
Middle East/North Africa	22,579 (4.8)	36,664 (2.8)	0.10
South Asia	60,435 (12.8)	100,998 (7.7)	0.17
Sub-Saharan Africa	12,171 (2.6)	26,760 (2.0)	0.04
Western Nations/Europe	22,113 (4.7)	50885 (3.9)	0.04
Maternal comorbidities within 365 d before conception			
Mean (SD) number of aggregated diagnosis groups	9.7 (3.7)	8.3 (3.9)	0.38
Prepregnancy type 1 or type 2 diabetes mellitus	16,065 (3.4)	13063 (1.0)	0.16
Chronic hypertension	17,572 (3.7)	22215 (1.7)	0.12
Any prepregnancy cardiovascular disease	36,816 (7.8)	63984 (4.9)	0.12
Substance or tobacco use	19,719 (4.2)	62976 (4.8)	0.03
Use of noninvasive assisted reproduction	17,552 (3.7)	27,537 (2.1)	0.10
Use of invasive assisted reproduction	10,065 (2.1)	16,895 (1.3)	0.06
Maternal conditions arising between pregnancy conception and the index birth			
Preeclampsia	1175 (0.2)	2223 (0.2)	0.00
Gestational hypertension	2595 (0.6)	5529 (0.4)	0.03
Of the newborn			
Female	229,523 (48.7)	636,471 (48.7)	0.00
Mean (SD) gestational age at birth, wk	38.6 (2.0)	38.8 (1.9)	0.09
Preterm birth < 37 wks’ gestation	38,605 (8.2)	90,349 (6.9)	0.05
Mean (SD) birthweight, g	3309 (577)	3354 (564)	0.08
Maternal laboratory measures within 365 d before conception			
Mean (SD) serum creatinine concentration, umol/L	61.8 (10.4)	—	—
Mean (SD) eGFR, mL/min/1.73 m^2^	114 (13)	—	—
eGFR categories, mL/min/1.73 m^2^		—	—
< 30	37 (0.0)	—	—
30 to 44	94 (0.0)	—	—
45 to 59	346 (0.1)	—	—
≥ 60	470,765 (99.9)	—	—
Urine protein^b^			
Severe albuminuria	3823 (0.8)	3961 (0.3)	0.07
Moderate albuminuria	17,501 (3.7)	12,564 (1.0)	0.18
Normal/mild albuminuria	209,402 (44.4)	94,557 (7.2)	0.94

All data are presented as a number (%) unless otherwise indicated.

Abbreviations: eGFR, estimated glomerular filtration; IQR, interquartile range.

a A standardized difference > 0.10 is thought to be clinically important.

b Urine protein was known among 230,726 of 471,242 women who had a serum creatinine concentration measured, and 111,082 of 1,305,681 women who did not have a serum creatinine concentration measured.

**Figure 1:**
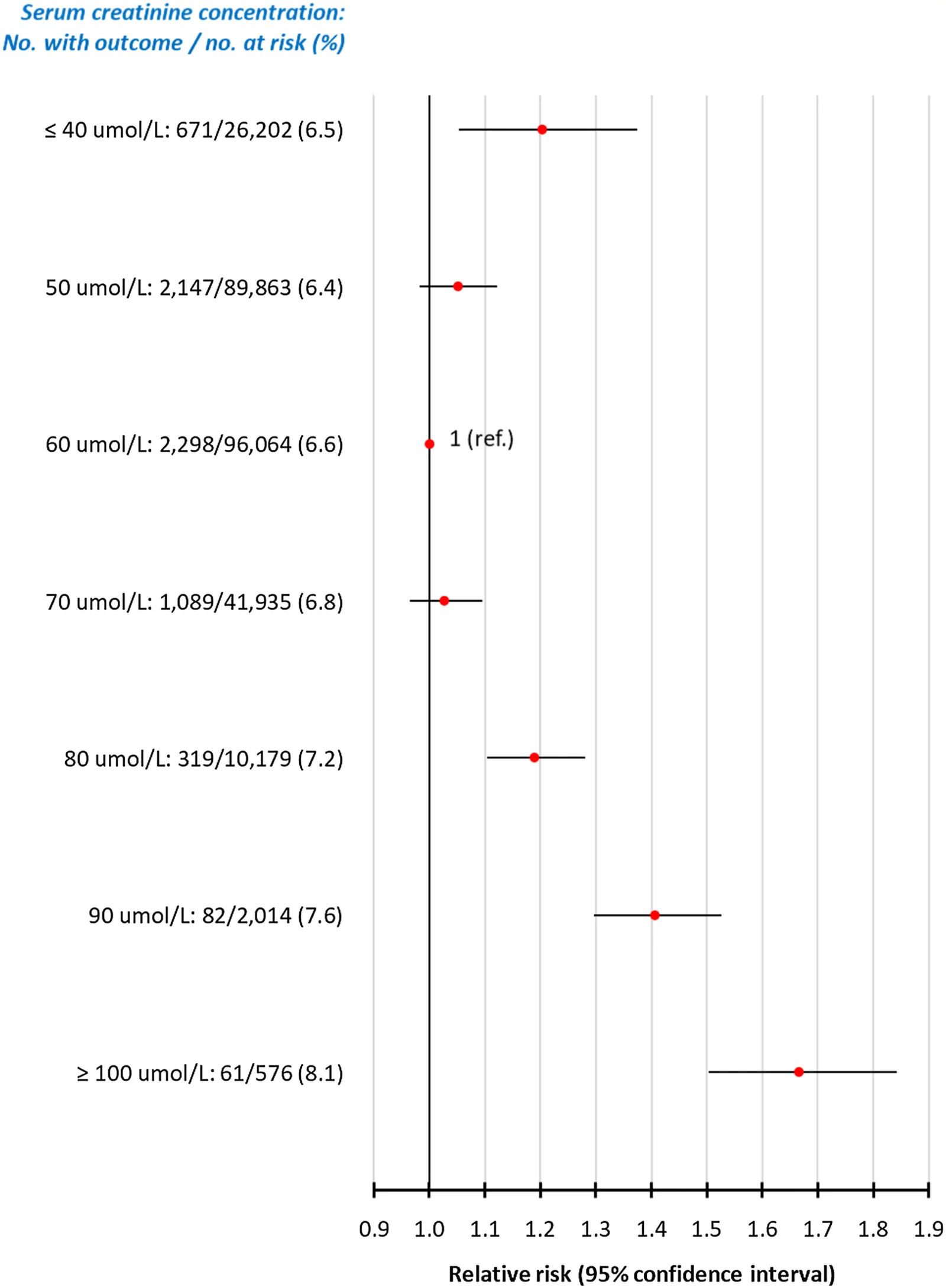
Risk of the composite outcome of severe maternal morbidity or mortality (SMM-M) occurring during pregnancy and up to 42 days after birth, in association with prepregnancy serum creatinine (SCr) concentration. Models were adjusted for the gestational age at which the SCr concentration was measured, maternal age, parity, rural or urban residence, residential income quintile, world region of origin, number of comorbidities, tobacco or substance use, as well as prepregnancy proteinuria.

Overall, SMM-M occurred among 6687 births (1.4%). There was a J-shaped relation seen between prepregnancy SCr concentration and SMM-M (Fig 1). For example, relative to an SCr concentration of 60 μmol/L (referent), the adjusted relative risk for SMM-M was 1.18 (95% CI, 1.11-1.28) at an SCr concentration of 80 μmol/L and 1.20 (95% CI, 1.05-1.38) at an SCr concentration of ≤ 40 μmol/L. Adjusting for body mass index yielded similar results (relative to an SCr concentration of 60 μmol/L [referent], the adjusted relative risk for SMM-M was 1.25 [95% CI, 1.11-1.40] at an SCr concentration of 80 μmol/L and 1.15 [95% CI, 1.05-1.25] at an SCr concentration of ≤ 40 μmol/L).

In this population-based study, prepregnancy SCr concentration demonstrated a J-shaped relation with subsequent SMM-M, even after accounting for maternal demographic and comorbidity measures. Although the relative risks of SMM-M across prepregnancy SCr concentrations were modest, the absolute number of affected births was substantial (1.4% of the cohort), indicating that even small relative increases may be clinically meaningful at the population level.

Interestingly, women with a low prepregnancy SCr concentration measurement demonstrated an increased risk of SMM-M. Given the relationship between SCr concentration and muscle mass, low values of this analyte may reflect underlying physiologic vulnerability imparted by chronic illness or poor nutritional status. Hence, such women may be less able to tolerate the hemodynamic and metabolic stresses of pregnancy, increasing their vulnerability to complications leading to SMM-M.

As a limitation, prepregnancy SCr concentration was ordered on clinical grounds or as part of a routine health check-up, so the cohort may partly include women at higher risk of developing SMM once pregnant. Even so, 99.9% of the cohort had an estimated glomerular filtration rate of > 60 mL/min/1.73 m^2^—a nonpathologic cut-point. Additionally, the rates of hypertensive disorders of pregnancy may have been undercaptured in our study, which could introduce nondifferential misclassification and modestly affect effect estimates.

Given its widespread availability, standardization, and low cost, prepregnancy (or early first-trimester) SCr concentration requires further validation as a marker of adverse maternal, obstetric, and perinatal outcomes.
