# Supplementary material for: Prepregnancy Serum Creatinine Concentration and Risk of Severe Maternal Morbidity and Mortality
Source: Kidney Med. 2026 Jun 25;8(10):101455. doi: 10.1016/j.xkme.2026.101455 (PMC13545362; doi:10.1016/j.xkme.2026.101455)
Supplement: Supplementary file 1 [file mmc1.pdf]

Supplementary Material

Figure S1: Cohort flow diagram

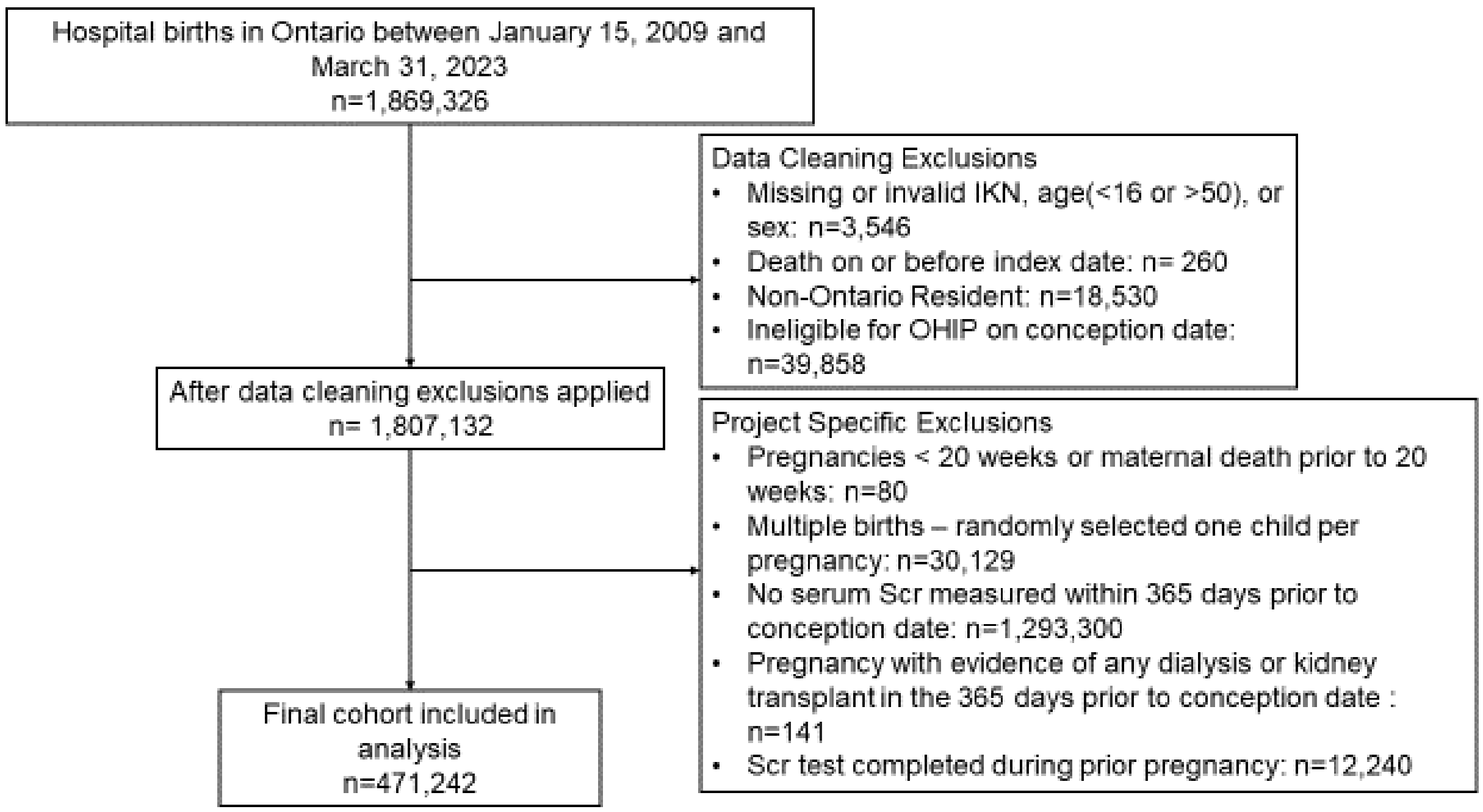

Table S1: List of ICES linked databases used in the current study

**Dataset name**	**Description**
The Better Outcomes Registry & Network (BORN)	Provincial body responsible for collecting pregnancy and newborn information and outcomes data for all births in Ontario, including enhanced First Trimester Screening.
Canadian Institute for Health Information Discharge Abstract Database (CIHI-DAD)	Captures all in-patient hospital admission records including obstetric deliveries and deaths. Diagnostic codes are based on the *International Statistical Classification of Diseases and Related Health Problems, Tenth Revision, Canada* (*ICD-10-CA*), and procedural codes are based on the *Canadian Classification of Health Interventions (CCI)*.
Immigration, Refugees and Citizenship Canada Permanent Residents Database (IRCC-PRD)	Captures demographic information on all international migrants who obtained permanent residency in Canada from January 1985 to March 2023.
Linked Delivering Mothers and Newborns (MOMBABY)	Derived from CIHI-DAD, provides linked inpatient hospital admission records of mothers and their infants.
National Ambulatory Care Reporting System (NACRS)	Collects data on hospital-and community-based ambulatory care, such as day surgery, outpatient and community-based clinics and emergency departments.
Ontario Health Insurance Plan Claims Database (OHIP)	Contains information about inpatient and ambulatory visits, consultations and procedures provided to Ontario residents eligible for Ontario's publicly funded health insurance system by fee-for-service health care practitioners (e.g., physicians, optometrists, laboratories for diagnostic tests etc.).
Ontario Laboratory Information Service (OLIS)	Contains laboratory data for inpatients and outpatients in Ontario.
Postal Code Conversion File Plus (PCCF+)	A digital file that links the Canada Post Corporation (CPC) six-character postal code and Statistics Canada's standard geographic areas (e.g., dissemination area). Area-level income quintiles range from Q1 (lowest) to Q5 (highest) income neighbourhoods.
Registered Persons Database (RPDB)	Includes vital status and sociodemographic information about all individuals who have ever received an Ontario Health Insurance Plan (OHIP) number (e.g., date of birth, sex, and postal code).
Same Day Surgery (SDS)	Contain demographic, diagnostic, procedural and treatment information about all day surgical procedures.
Statistic Canada Census	Information from the Canadian Census, statistical information about the population including population

Table S2: Cohort entry and exclusion criteria, methods, and coding to identify study outcomes

Assessment	Timing	Disease, procedure or condition	ICD-10-CA or CCI codes in CIHI-DAD, SDS and NACRS	Diagnostic & fee codes in OHIP	Other sources
Inclusion criteria	January 15, 2009 to March 31, 2023	• All hospital singleton livebirths and stillbirths at $20^{0/7}$ to $42^{0/7}$ weeks' gestation.<br>• Births limited to women aged 16 to 50 years old with a valid OHIP/IKN number from MOMBABY.<br>• Stillbirth: fetal death arising *in utero*, or a newborn with no signs of life at birth, at ≥ 20 weeks' gestation -- among livebirths and stillbirths.	MOMBABY	--	--
	April 1, 2007 to March 31, 2022	Serum creatinine concentration measurement within 365 days before conception	--	--	OLIS
Exclusion criteria	At the mother's index delivery hospitalization for the first & second births	Records with warning for mother's IKN or KEY	MOMBABY/RPBD: warning for IKN/KEY (WARN not =”N”(No Warning)). Include N=no warning	--	--
	At the mother's index delivery hospitalization for the first & second births	Infant gestational age at birth < 20 or ≥ 43 weeks' gestation or gestational age missing	MOMBABY:<br>a. B_GESTWKS_DEL not in MOMBABY<br>b. gestational age <20 or ≥43 weeks' gestation	--	--
	At the mother's index delivery hospitalization for the first & second births	Women aged < 16 or > 50 years, or age missing	MOMBABY/RPDB:<br>M_IKN age < 15 or > 50 or missing	--	--

Assessment	Timing	Disease, procedure or condition	ICD-10-CA or CCI codes in CIHI-DAD, SDS and NACRS	Diagnostic & fee codes in OHIP	Other sources
	Same as above	Women who had an invalid OHIP number or hospital number	MOMBABY/RPDB:<br>Invalid M_IKN (maternal IKN)<br>a. VALIKN ne 'V'<br>b. M_IKN not in RPDB (according to no sex and no bdate)<br>c. M_IKN with sex ='M' in RPDB	--	--
	Same as above	Women ineligible for OHIP	--	--	RPDB
	Same as above	Women who were a non-Ontario resident	--	--	RPDB: M_IKN non-Ontario resident (substr (prcddablk, 1,2 ne '35')
Main exposure	Up to 365 days prior to conception	Serum creatinine concentratio	--	--	OLIS

Assessment	Timing	Disease, procedure or condition	ICD-10-CA or CCI codes in CIHI-DAD, SDS and NACRS	Diagnostic & fee codes in OHIP	Other sources
Main outcome	From 20 weeks' gestation and up to 42 days after the index birth hospitalization discharge date.<br><br>NB: If the birth hospitalization length of stay was ≤ 42 days, then SMM-M was further assessed within any subsequent rehospitalization, up to 42 days after birth.<br><br>If the birth hospital duration was > 42 days, then SMM-M was assessed any time during that hospitalization, even if it surpassed 42 days.	Severe maternal morbidity (SMM) – among livebirths & stillbirths	Severe preeclampsia and (HELLP) syndrome:<br>O14.1, or O14.2<br><br>Eclampsia:<br>O15<br><br>Cerebral venous thrombosis in pregnancy, or in the puerperium:<br>O22.5, or O87.3<br><br>Acute fatty liver with red blood cell (RBC) transfusion or plasma transfusion:<br>O26.6 + (CIHI BTREDBC = 1 or CIHI BTPLASMA = 1)	--	Based on validated composite of SMM-M, developed by the Canadian Perinatal Surveillance System[a]

Assessment	Timing	Disease, procedure or condition	ICD-10-CA or CCI codes in CIHI-DAD, SDS and NACRS	Diagnostic & fee codes in OHIP	Other sources
			Pulmonary, cardiac, and CNS complications of anesthesia during pregnancy, the puerperium or labour and delivery:<br>O29.0, O29.1, O29.2, O89.0, O89.1, O89.2, O74.0, O74.1, O74.2 or O74.3<br><br>Placenta previa with hemorrhage with RBC transfusion:<br>O44.1 + CIHI BTREDBC = 1<br><br>Placental abruption with coagulation defect:<br>O45.0<br><br>Antepartum hemorrhage with coagulation defect:<br>O46.0<br><br>Intrapartum hemorrhage with coagulation defect:<br>O67.0<br><br>Intrapartum hemorrhage with RBC transfusion:<br>O67 + CIHI BTREDBC = 1		

Assessment	Timing	Disease, procedure or condition	ICD-10-CA or CCI codes in CIHI-DAD, SDS and NACRS	Diagnostic & fee codes in OHIP	Other sources
			<u>Rupture of the uterus with RBC transfusion, procedures to the uterus or hysterectomy:</u><br>• (O71.0 or O71.1) + any of the following: CIHI BTREDBC = 1, <u>or</u><br>• (1.RM.13, 1.KT.51, 5.PC.91.LA or 5.PC.91.HV) + CIHI BTREDBC = 1, <u>or</u><br>• (5.MD.60.RC, 5.MD.60.RD, 5.MD.60.KE, 5.MD.60.CB or 1.RM.89.LA[a]), <u>or</u><br>• 1.RM.87.LA-GX<br>[a]**NOTE**: **1.RM.89.LA** is included only if codes 1.PL.74, 1.RS.74 or 1.RS.80 are NOT also present<br><br><u>Postpartum hemorrhage with RBC transfusion, procedures to the uterus or hysterectomy:</u><br>O72 + any of the following:<br><br>BTREDBC = 1, <u>or</u><br>• (1.RM.13, 1.KT.51, 5.PC.91.LA or 5.PC.91.HV) + BTREDBC = 1, <u>or</u><br>• (5.MD.60.RC, 5.MD.60.RD, 5.MD.60.KE, 5.MD.60.CB or 1.RM.89.LA[b]), <u>or</u><br>• 1.RM.87.LA-GX<br>[b]**NOTE**: **1.RM.89.LA** is included only if codes 1.PL.74, 1.RS.74 or 1.RS.80 are NOT also present<br><u>Cardiac conditions:</u><br>O74.2, O89.1, O90.3, I21, I22,		

Assessment	Timing	Disease, procedure or condition	ICD-10-CA or CCI codes in CIHI-DAD, SDS and NACRS	Diagnostic & fee codes in OHIP	Other sources
			Obstetric shock:<br>O75.1, R57, T80.5 or T88.6<br><br>Septicemia during labour:<br>O75.3<br><br>Complications of obstetric surgery and procedures:<br>O75.4<br><br>Puerperal sepsis:<br>O85<br><br>Obstetric embolism:<br>O88<br><br>Acute renal failure:<br>O90.4, N17, N19 or N99.0<br><br>Disseminated intravascular coagulation:<br>D65<br><br>Sickle cell anemia with crisis:<br>D57.0<br><br>Acute psychosis:<br>F53.1 or F23<br><br>Status epilepticus:<br>G41		

Assessment	Timing	Disease, procedure or condition	ICD-10-CA or CCI codes in CIHI-DAD, SDS and NACRS	Diagnostic & fee codes in OHIP	Other sources
			<u>Cerebrovascular diseases: subarachnoid and intracranial hemorrhage, cerebral infarction, stroke:</u><br>I60, I61, I62, I63 or I64<br><br><u>Status asthmaticus:</u><br>J45.01, J45.11, J45.81 or J45.91<br><br><u>Adult respiratory distress syndrome</u>:<br>J80<br><br><u>Acute abdomen:</u><br>K35, K37, K65, N73.3 or N73.5<br><br><u>Hepatic failure:</u><br>K71 or K72<br><br><u>Assisted ventilation through endotracheal tube:</u><br>1.GZ.31.CA-ND<br><br><u>Assisted ventilation through tracheostomy:</u><br>1.GZ.31.CR-ND<br><br><u>Hysterectomy</u>:<br>5.MD.60.RC, 5.MD.60.RD, 5.MD.60.KE, 5.MD.60.CB, 1.RM.89.LA (exclude if 1.PL.74, 1.RS.74 or 1.RS.80 code also		

Assessment	Timing	Disease, procedure or condition	ICD-10-CA or CCI codes in CIHI-DAD, SDS and NACRS	Diagnostic & fee codes in OHIP	Other sources
			Evacuation of incisional hematoma with RBC transfusion:<br>5.PC.73.JS + CIHI BTREDBC = 1<br><br>Repair of bladder, urethra, or intestine:<br>5.PC.80.JR, 1.NK.80, 1.NM.80<br><br>Procedures to the uterus/pelvic vessels with RBC transfusion:<br>(1.RM.13, 1.KT.51, 5.PC.91.LA, 5.PC.91.HV) + CIHI BTREDBC = 1<br><br>Surgical or manual correction of inverted uterus for vaginal births only:<br>5.PC.91.HQ or 5.PC.91.HP, restricted to vaginal births (i.e., absence of caesarean 5.MD.60)<br><br>Reclosure of caesarean wound with RBC transfusion:<br>(5.PC.80.JM, 5.PC.80.JH) + CIHI BTREDBC = 1<br><br>Curettage with RBC transfusion:<br>(5.PC.91.GA, 5.PC.91.GC, 5.PC.91.GD) + CIHI BTREDBC = 1<br><br>Maternal ICU admission:<br>SCU in (‘10’, ’20’, ’25’, ’30’, ’35’, ’40’,’45’,’60’,’80’)		

[a]Dzakpasu S, Deb-Rinker P, Arbour L, et al. Severe maternal morbidity surveillance: Monitoring pregnant women at high risk for prolonged hospitalisation and death.Paediatr Perinat Epidemiol. 2020;34(:427-439.

Abbreviations: ACG: Adjusted Clinical Group; ADG: Aggregated Diagnosis Group; CIHI: Canadian Institute for Health Information; DAD: Discharge Abstract Database; CCI: Canadian Classification of Health Interventions; ICD-10-CA: International Classification of Diseases, 10th Revision, Canada; IRCC-PRD: Immigration, Refugees, and Citizenship Canada Permanent Resident Database; NACRS: National Ambulatory Care Reporting System; OHIP: Ontario Health Insurance Plan; PCCF+: Postal code conversion file plus (Statistics Canada); RPDB: Registered Persons Database; SDS: Same Day Surgery Database.
